# Molecular Study and Phylogenetic Analysis of Middle East Respiratory Syndrome Corona Virus (MERSCoV) in Camel and Human

**DOI:** 10.1088/1742-6596/1294/6/062097

**Published:** 2019-09

**Authors:** Saba F Alsalihi, Alaa Abdelkadhim Jawad, Mohsen A Al-Rodhan

**Affiliations:** 1 University of Al-Qadisiyah- College of Veterinary Medicine, Zoonotic diseases Unit. saba.ffff@gmail.com; 2 University of Al-Qadisiyah- College of Veterinary Medicine, Zoonotic diseases Unit.; 3 University of Al-Qadisiyah- College of Pharmacy

## Abstract

Middle East Respiratory Syndrome Corona Virus (MERSCoV) have been reported in Arabian peninsula and sporadic cases in Europe and Asia. This study was conducted to evaluate the genetic analysis of this virus in human and camel at the first time in Iraq. Two hundred samples were collected from camels and human who suffering from respiratory symptoms, these samples treated with RNA extraction kit then amplification the genetic material by PCR which give 5% positive results. The amplicon then sequenced, registration in gene bank of NCBI for getting accession numbers. The local strains give close relationship with neighbor countries as Saudi Arabia and Jordan strains when using MEGA analysis software.

## Introduction

Most of Middle East Respiratory Syndrome cases have been reported in Arabian Peninsula in addition to document of sporadic cases in Europe and Asia. Genetic recombination implicated in the emergence of (MERSCoV), virulence host adaptation, transmission and other zoonotic and epidemiological features, this study was conducted to evaluate the genetic relationship among Middle East respiratory syndrome coronavirus (MERSCoV) of human and camels' origin at the period from October 2015 to February 2016. Hundred samples were collected from camel and 100 from human. Camel,Human and camel samplescarried out by RNA extraction100 human nasal swaps and bronchial lavage samples were collected from pilgrims and non-pilgrims, the total positive result was 5%. The pilgrims recorded the highest infection rate. The result of conventional PCR for detection of Ngene (217 bp) of MERSCoV was confirmative, three humans and 11 camel positive samples were used in further sequencing and phylogenetic analysis by extraction and purification of the PCR products. Our clones sequence submitted in GenBank-NCPI and took their accession number.

(Camel- KX150506.1, KX150493.1, KX150494.1, KX150495.1, KX150496.1, KX150497.1, KX150498.1, KX150502.1, KX150503.1, KX150504.1, KX150505.1), (Human- KX150499.1, KX150500.1, KX150501.1the phylogenetic tree construction and analysis resultsshowed that most of Iraqi

Content from this work may be used under the terms of the Creative Commons Attribution 3.0 licence. Any further distribution of this work must maintain attribution to the author(s) and the title of the work, journal citation and DOI.
Published under licence by IOP Publishing Ltd

IOP Conf. Series: Journal of Physics: Conf. Series **1294** (2019) 062097 doi:10.1088/1742-6596/1294/6/062097

variants of camel and human were located in Clade-B in which Saudi Arabia strains were clustered. One of our clones (MERS-Iq.2Huh) of accession number KX150500.1 was located in clade-A in the same branch of Jordanian strain. Middle East Respiratory Syndrome Corona Virus (MERSCoV) is widely spread in Arabian Peninsula and many other Middle East countries surrounded of Iraq [1, 2,3,4,5]. It has become one of the most important emerging human health threating virus [6].MERSCoV is beta corona virus within coronaviridaefamily which are enveloped, positive sense RNA genome with nucleocapsid of helical symmetry infect human and variety of animal species [7]. The ability of high recombination, unique viral replication and low fidelity of corona virus polymerases allows for unexpected viral evolution to infect other host.[8,9 ] Phylogenetic analysis of African bat virus belonging to the same species of MERSCoV and indicate that the evolution of the virus in camels precede that in human suggesting the possible spreading from bats to camels took place in Africa and involved exchange of genetic materials among ancestral virus strains[10,11]Nucleocapsid gene (N gene) is common target for cloning phylogenetic analysis and generation recombinants portions. N protein is highly immunogenic phosphoprotein and modulation of cell signaling method. The N gene has been used for corona virus genotyping and phylogenetic analysis which helped our knowledge of virus temporal geographic origins and evolution [12,13]. This study was conducted to evaluate the genetic relationship among local circulating MERSCoV variants in human and camel at the first time in Iraq.

## Materials and methods

This study was carried out by collection of 100 nasal and oropharyngeal swap samples from camels and 100 nasal swap and bronchial lavage samples from humans at the period from October 2015 to February 2016 from both sexes and different ages in many locations of Middle Euphrates/IraqViral RNA has been extracted by Total RNA Extraction KitAccuZol™ kit bioneer Korea. The extracted RNA has been measured for concentration and purity by Nanodrop. Reverse transcription conducted by AccuPower® Rocket Script™ RT PreMix 96 plate kit bioneer Korea Conventional end point PCR for detection of *N gene* fragment 217 bp, the PCR products of positive samples were extracted and purified and sequenced by dye-terminator based sequenced illumina by AB. The genomic sequences were assembled and submitted in GenBank- NCBI then multiple sequence alignment was done by clustal Omega for phylogenetic tree construction and phylogenetic analysis [12, 17]. PCR for detection of Ngene (217 bp) of MERSCoV by using specific primers (F- TGCAAGCTTTTGGTCTTCGC), (R-ATGGCTCCACTGTACCGAAG)

Statistical analysis using Chi square to assess statistical significance according to [18]

## Results

The result of conventional PCR of camel and human were confirmative Fig.-1, Fig.-2. Fourteen of our clones which were 11 of camel and 3 of human were took their accession number in GenBank-NCBI.

The result of phylogenetic analysis showed most of Iraqi clones were grouped in clad B and clustered mainly with the same branch of Saudi Arabia and South Korea, only one clone in this study clustered in the same branch of Jordanian in clade A. while *bat corona virus*, *SARSV* and *neoromica corona virus* was out group clustered in separated branch

IOP Conf. Series: Journal of Physics: Conf. Series **1294** (2019) 062097 doi:10.1088/1742-6596/1294/6/062097

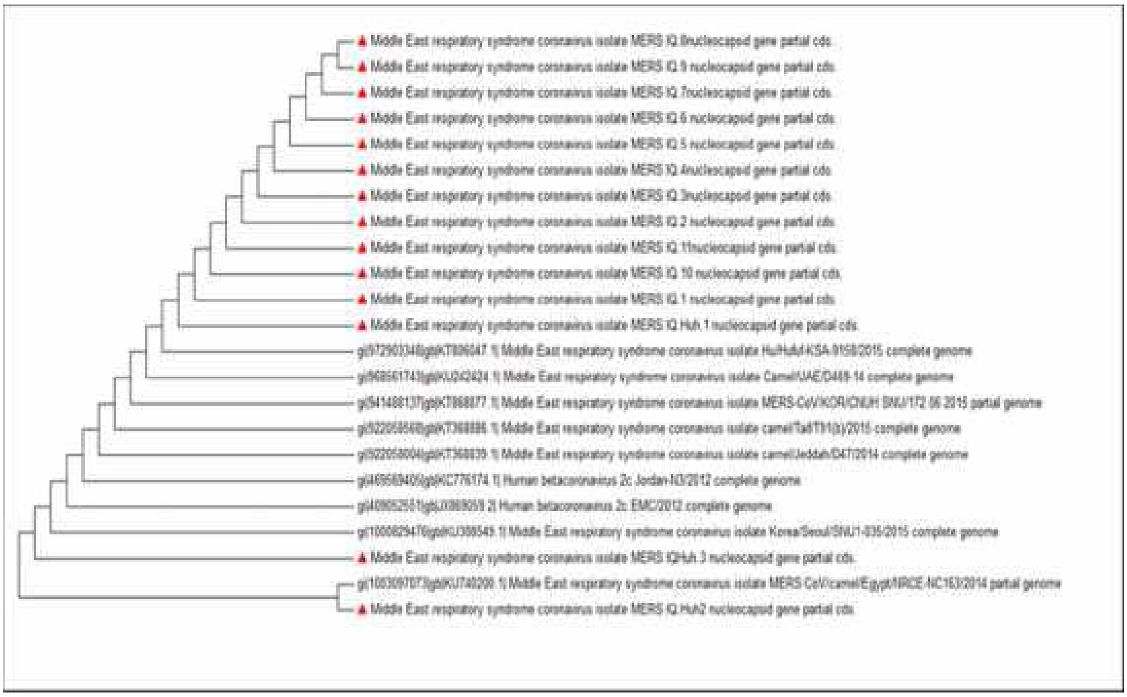

## Discussion

*MERSCoV*was first recorded in 2012 in Saudi Arabia, the virus associated with severe respiratory illness, renal failure and high rate of mortality 50% [18, 19]. The emergence of this important infectious pathogen has raised global concerns regarding the current epidemiological features and its future evolution. Camels may consider the potential source of human infection and act as reservoir transmit the virus to human. This study was designed for molecular characterization of the virus and to explain some epidemiological of MERS [20, 21].

Phylogenetic analysis demonstrates that most Iraqi variants of *MERSCoV* of camel and human of this study fell in the Clade-B with Saudi strains as well as 2015 South Korea outbreak strains [31 ,32, 17] due to continuous mixing and introduction of camel across Saudi borders in addition to large number of travelers in Hajj season. Furthermore, recent recombination and emergence of that novel virus play a role in close relation and high identity among these strains of *MERSCoV*.

## Conclusions

MERS was widely distributed in apparently healthy camels at the western borders of Iraq, the risk of pilgrims returning with *MERSCoV* during the large mass gathering of annual Hajj and minor Umrah must be considered. Genotypically, human and camel variants fell in the same branch of phylogenetic tree with about 100% identity that indicates the role of camel as a reservoir or intermediate host in zoonotic transmission. Application RT-RT-PCR screening technique in combination with wide seroepidemiological surveys for the disease in aiding in the strict surveillance.

## References

[1] Zaki AM, van Boheemen S, Bestebroer TM (2012). Isolation of a novel coronavirus from a man with pneumonia in Saudi Arabia. N. Engl. J. Med..

[2] Reusken CB, Farag EA, Jonges M (2014). Middle East Respiratory Syndrome Coronavirus (MERS-CoV) RNA and neutralising antibodies in milk collected according to local customs from dromedary camels, Qatar, Eurosurveillance.

[3] Alexandersen S, Kobinger GP, Soule G, Wernery U (2014). *Middle East respiratory Syndrome Coronavirus* antibody reactors among camels in Dubai, United Arab Emirates, in 2005. Transbound. Emerg. Dis.;.

[4] Reusken CB, Ababneh M, Raj VS (2013). *Middle East Respiratory Syndrome coronavirus (MERS-CoV*) serology in major livestock species in an affected region in Jordan, June to September. Eurosurveillance:.

[5] Alagaili AN, Briese T, Mishra N (2014). *Middle East Respiratory Syndrome Coronavirus* infection in dromedary camels in Saudi Arabia. MBio;.

[6] Alqabandi S, Al-Qasser, Madi (2013). Detection of MER circulation in Kuwait. Infec. dis. &Threapy.

[7] Shehata MM, Gomma Mr, Ali AA and Ghai K MERSCoV comprehensive review in Frontiers of Medicine.www.Researchgate.net, 2012

[8] Zhang Z., Shen, L &Gu X. (2016). Evolutionary dynamics of MERS-CoV: Potential Recombination, positive Selection and transmission.

[9] Kandil A, Shehata MM, ElShesheny R, Gomma MR, Ali MA (2016). Kayali Complete genome sequences of *MERSCoV* isolated from dromedary camel in Egypt. Am. Society for Microbiology.

[10] Chastel C. (2014). Middle East respiratory syndrome (MERS): bats or dromedary, which of them is responsible?. Bull SocPatholExot.

[11] WHO). Middle East respiratory syndrome coronavirus (MERS-CoV) Available at: www.who.intemergencies/mers/en/ [accessed January 28, 2016]

[12] Cotten M, Lam TT, Watson SJ, Palser AL, Petrova V, Grant P (2013). Full-genome deep sequencing and phylogenetic analysis of novel human *betacoronavirus*. Emerg. Infect. Dis..;.

[13] vanBoheemen S, de Graaf M, Lauber C, Bestebroer TM, Raj VS, Zaki AM (2012). Genomic characterization of a newly discovered coronavirus associated with acute respiratory distress syndrome in humans. MBio..

[14] Song D, Ha G, Serhan W, Eltahir Y (2015). Development and validation of a rapid immunochromatographic assay for detection of *Middle East Respiratory Syndrome Coronavirus* antigen in dromedary camels. J ClinMicrobiol.

[15] Mackay IM, Arden K E. (2015). MERSCoV: diagnostics, epidemiology and transmission. Virology Journal.

[16] Bhadra S, Jiang YS, Kumar MR (2015). Real time sequence-validated loop-mediated isothermal amplification assays for detection of *Middle East Respiratory Syndrome Coronavirus (MERS-CoV*). PLoS One.;.

[17] Kim Y, Cheon S, Min C, Sohn KM (2016). Spread of Mutant *Middle East Respiratory Syndrome Coronavirus* with Reduced Affinity to Human CD26 during the South Korean Outbreak. Am.Society for Microbiology.

[18] Hemida MG, Chu DK, Poon LL (2014). *MERSCoV* in dromedary camel herd, Saudi Arabia. Emerg. Infect. Dis;.

[19] Yusof MF, Eltahir YM, Serhan WS, Hashem FM, et al. Prevalence of Middle East Respiratory Syndrome

[20] Coronavirus (MERS-CoV) in dromedary camels in Abu Dhabi Emirate, United Arab Emirates [cited 2015 Feb Virus Genes. 2015 [Epub ahead of print] http://www.ncbi.nlm.nih.Gov/PubMed/25653016, 2015: Feb 5 10.1007/s11262-015-1174-0 PMC7088545 25653016

[21] Hemida MG, Perera RA, Al Jassim RA, et al. Seroepidemiology of Middle East Respiratory Syndrome

[22] (2014). (*MERSCoV*) in Saudi Arabia and Australia (2014) and characterisation of assay specifi city. Eurosurveillance.

[23] Chu DK, Poon LL, Gomaa MM (2014). *MERSCoV* in dromedary camels. Egypt. Emerg Infect Dis.

[24] Perera RA, Wang P, Gomaa MR (2013). Seroepidemiology for *MERSCoV*using microneutralisation and pseudoparticle virus neutralisation assays reveal a high prevalence of antibody in dromedary camels in Egypt, June 2013. Eurosurveillance.

